# Home exercise therapy to improve muscle strength and joint flexibility effectively treats pre-radiographic knee OA in community-dwelling elderly: a randomized controlled trial

**DOI:** 10.1007/s10067-018-4263-3

**Published:** 2018-08-30

**Authors:** Yusuke Suzuki, Hirotaka Iijima, Yuto Tashiro, Yuu Kajiwara, Hala Zeidan, Kanako Shimoura, Yuichi Nishida, Tsubasa Bito, Kengo Nakai, Masataka Tatsumi, Soyoka Yoshimi, Tadao Tsuboyama, Tomoki Aoyama

**Affiliations:** 1 Department of Physical Therapy, Human Health Sciences, Graduate School of Medicine, Kyoto University, 53 Kawahara-cho, Shogoin, Sakyo-ku, Kyoto, 606-8507 Japan; 2 Department of System Design Engineering, Keio University, Yokohama, Japan

**Keywords:** Home exercise, Knee osteoarthritis, Muscle, Randomized controlled trial, Stretching, Training

## Abstract

To compare the efficacy and adherence rates of two parallel home exercise therapy programs—multiple exercise (training and stretching the knee and hip muscles) and control (training the quadriceps muscles)—on knee pain, physical function, and knee extension strength in community-dwelling elderly individuals with pre-radiographic knee osteoarthritis (OA). One hundred patients with medial knee pain were randomly allocated to one of two 4-week home exercise programs. Individuals with a Kellgren/Lawrence (K/L) grade 0 or 1 OA (pre-radiographic knee OA) in the medial compartment were enrolled. Primary outcomes were knee pain (visual analog scale), self-reported physical function (Japanese Knee Osteoarthritis Measure [JKOM]), and isometric maximum muscle strength of the knee extensor measured using a hand-held dynamometer. A total of 52 patients (28 [53.8%] in the multiple exercise group, 24 [46.2%] in the control group) completed the trial. The JKOM activities of daily living and general health conditions outcomes improved significantly in the multiple exercise group compared to the control group (JKOM activities of daily living, beta = − 0.76; 95% confidence interval [CI], − 1.39 to − 0.13; *p* = 0.01; JKOM general health conditions, beta = − 0.25; 95% CI, − 0.48 to − 0.01; *p* = 0.03). The home exercise compliance rates of the multiple exercise and control groups were 96.6 and 100%, respectively. When targeting pre-radiographic knee OA in community-dwelling elderly, it is important to implement home exercise programs that aim to improve muscle strength and joint flexibility rather than knee extension muscle power only.

## Introduction

Knee osteoarthritis (OA), a painful disease involving degeneration of the articular cartilage, has a prevalence in Japanese people > 60 years of age of 47.0% in men and 70.2% in women, making it a common disease [1]. Worsening knee OA severity leads to decreased activity limitations, creating the need to prevent its onset or progression [2]. However, effective treatment and preventive methods have not been established, and symptomatic treatment is the main treatment method.

Kellgren/Lawrence (K/L) classification by radiographic evaluation of knee OA is generally performed, and grade increases as joint deformity severity increases [3]. However, there is reportedly no clear relationship between K/L classification and subjective pain [4] or between K/L classification result and thigh muscle strength or functional capacity [5, 6]. Even in clinical practice, some subjects have knee pain and dysfunction despite a low K/L classification (pre-radiographic knee OA). Thus, when considering the purpose of preventing and treating knee OA in community-dwelling elderly individuals, rather than target a K/L grade ≥ 2 with knee OA as per the conventional definition, it is considered important to target a K/L grade of 0 or 1.

Non-pharmacotherapy such as patient education, weight loss, and exercise are recommended, although knee OA treatments are broadly classified into drug therapy and non-drug therapy [7]. Among non-pharmacotherapy regimens, exercise therapy has proven effective in previous studies [8]. Strength training of the quadriceps muscles in particular was shown in an intervention study and systematic review to effectively relieve pain and improve physical function [9, 10]. On the other hand, muscle strength of the hip is significantly reduced in patients with knee OA compared with healthy subjects [11], and exercise therapy of the hip muscles seems important for knee OA.

Besides muscle strength, range of motion (ROM) limitations are strongly related to knee OA-induced dysfunction [12]. Previous studies reported that the hamstring activity of patients with knee OA is higher than usual, which is associated with decreased ROM of the knee extensors during walking and further ROM restrictions of the knee joint [13, 14].

Therefore, the planning of exercise therapy for knee OA must consider a combination of various factors such as strengthening the knee and hip joint muscles and improving knee joint flexibility.

Exercise therapy is classified into supervised programs and at-home programs. The latter is inexpensive and does not require special equipment, and with full patient adherence, can achieve the same effect as supervised exercise therapy [15]. Therefore, home exercise programs are considered effective for treating and preventing knee OA progression in community-dwelling elderly individuals.

However, complicated exercises that are difficult for the subject to understand lead to adherence obstacles [16]. Therefore, a program to improve the strength of multiple muscles and increase joint flexibility often provides simple muscle strength training of the quadriceps. On the other hand, the individualization of exercise programs is reportedly associated with high adherence rates [17]. For this reason, if it is possible to provide individualized exercise components that a subject can easily understand, the adherence rate will likely be high and the program will improve the strength of multiple muscles and increase joint flexibility.

Therefore, the hypothesis of this study is that (1) home exercise therapy that aims to increase the strength of multiple muscles and improve joint flexibility is more effective than targeted quadriceps muscle strengthening; and (2) adherence to home exercise therapy that aims to improve the strength of multiple muscles and increase joint flexibility will be preserved if participants are provided exercises with easy-to-understand and individualized content. However, no studies to date have reported on the efficacy of home exercise therapy for improving the strength of multiple muscles and increasing joint flexibility in a community-dwelling group of elderly individuals with pre-radiographic knee OA. Thus, this study aimed to (1) investigate the efficacy of home exercise therapy to improve the strength of multiple muscles and increasing joint flexibility in community-dwelling elderly individuals with pre-radiographic knee OA; and (2) examine the adherence rates to such home exercise programs.

## Materials and methods

### Study design

This single-participant blinded RCT and pre-post design comparing two parallel groups—multiple exercise (training and stretching the knee and hip muscles) and control (training the quadriceps muscles only)—was conducted between September and October 2017.

### Participants

Elderly participants reporting current knee pain were identified through a mailed survey and invited to visit the research facility at Kyoto University in September and October 2017. The ethical committee of Kyoto University approved the study (approval number, C1297), and written informed consent was obtained from all participants prior to enrollment. All recruited participants had a history of uni- or bilateral knee pain. The eligibility criteria included (1) age ≥ 45 years; (2) pre-radiographic knee OA (i.e., K/L grade 0 or 1 according to the original version [3] in one or both knees in the medial tibiofemoral compartment evaluated on weight-bearing anteroposterior radiographs); and (3) ability to walk independently on a flat surface without an ambulatory assistive device. Bilateral knee OA cases were not considered separately from unilateral cases. The exclusion criteria included (1) history of knee surgery; (2) rheumatoid arthritis; (3) periarticular fracture; (4) present neurological problems; or (5) mild or severe radiographic knee OA (i.e., K/L grade 2–4).

### Exercise interventions

All participants were taught a home exercise program by a physical therapist. An exercise instruction booklet was supplied to the participants to increase their understanding of the programming. Individuals allocated to the multiple exercise group were trained to perform 3 of the following 10 programs: (1) chair-sitting isotonic exercise for the quadriceps muscle; (2) isometric exercise for the quadriceps muscles; (3) supine positioning isotonic exercise for the hip extension muscles; (4) chair-sitting isometric exercise for the hip adduction muscles; (5) supine positioning isotonic exercise for the hip abduction muscles; (6) side-lying isotonic exercise for the hip abduction muscles; (7) narrow stance squat; (8) wide stance squat; (9) chair-sitting stretch for the hamstrings; and (10) side-lying stretch for the quadriceps muscles. Individuals allocated to the control group were trained to perform a single program: chair-sitting isotonic exercise for the quadriceps muscle (Table 1). The exercise program for participants in the multiple exercise group was decided based on the results of the interview conducted while the physical therapists assessed the participants’ clinical symptoms. The participants were instructed to perform the exercise program 5 times per week for 4 weeks and complete 10 repetitions per set for 3 sets of each exercise to the point of fatigue. All exercises were performed for one leg with knee pain. We investigated adherence by referring to daily exercise calendars on which the participants recorded their exercise frequency.

**Table 1:** Summary of exercise programs

(1) Chair-sitting isotonic exercise for the quadriceps muscle	Sit in a chair with the knees at 90° of flexion, then fully extend them using the resistance of one’s own weight.
(2) Isometric exercise for the quadriceps muscles	Sit with the knees extended. Contract the quadriceps muscle while elevating the heel and pushing the knee toward the mat.
(3) Supine positioning isotonic exercise for the hip extension muscles	Assume a supine position with the knees flexed and the hip fully up using the resistance of one’s own weight.
(4) Chair-sitting isometric exercise for the hip adduction muscles	Sit in a chair with the knees at 90° of flexion and a towel between the thighs. Use hip adduction to push the thigh toward the towel.
(5) Supine positioning isotonic exercise for the hip abduction muscles	In a supine position, perform isometric hip abduction using the resistance of one’s own weight.
(6) Side-lying isotonic exercise for the hip abduction muscles	In a side-lying position, perform isometric hip abduction using the resistance of one’s own weight.
(7) Narrow stance squat	Squat using a narrow stance.
(8) Wide stance squat	Squat using a wide stance.
(9) Chair-sitting stretch for the hamstrings	Sit in a chair with one knee extended. Tilt the upper body forward and stretch the hamstrings on the same side.
(10) Side-lying stretch for the quadriceps muscles	In a side-lying position with the knees flexed, pull one foot behind the body and stretch the quadriceps on the same side.

### Outcome measurements

For all participants, the following outcome measurements were evaluated: (1) demographic data; (2) radiographic evaluation; (3) knee OA-related health domain measure (the Japanese Knee Osteoarthritis Measure [JKOM]); and (4) measurements of lower-limb muscle strength.

### Demographic data

Data on age, sex, and height were self-reported by the patients. Weight was measured on a scale with the participants dressed but barefoot. Body mass index (BMI) was calculated by dividing the weight in kilograms by the squared height in meters.

### Radiographic evaluation

Anteroposterior radiographs of both knees in the fully extended weight-bearing and foot map positions were obtained at enrollment. The radiographic severity of the medial compartment in the tibiofemoral joint was assessed by a trained examiner. The K/L grade was scored as follows: 0 = normal; 1 = doubtful joint space narrowing (JSN) and possible osteophyte; 2 = definite osteophyte and possible JSN; 3 = multiple osteophytes, definite JSN, some sclerosis, and possible deformity of the bone ends; and 4 = large osteophyte, marked JSN, severe sclerosis, and definite deformity of the bone ends. The intra- and inter-rater agreements for K/L grade determination were excellent (intra-rater, κ = 0.88, 95% CI = 0.83, 0.92; inter-rater, κ = 0.84, 95% CI = 0.79, 0.90) [18].

### Knee pain severity and self-reported physical function

Knee pain severity and self-reported physical function were evaluated using the JKOM, a patient-based self-answered evaluation scoring system that assesses “pain and stiffness” (8 questions, 0–32 points), “activities of daily living” (10 questions, 0–40 points), “participation in social activities” (5 questions, 0–20 points), and “general health conditions” (2 questions, 0–8 points) with a maximum score of 100 points in a person-specific assessment [19]. The concurrent and construct validities of the JKOM were established by comparison of the Western Ontario and McMaster Universities Arthritis Index and the Medical Outcomes Study 36-item Short-Form Health Survey [19].

### Muscle strength

The maximum isometric knee extensor strengths in both legs were measured using a hand-held dynamometer (HHD) in accordance with the previously validated method for community-dwelling elderly fallers [20]. An HHD is a simple tool for objectively quantifying muscle strength that is widely used in clinical practice. Maximum force was recorded in Newtons (N) and 2 measurements were taken for each leg. The participants were instructed to remain seated in an upright position. The knee was placed at 90° flexion with the HHD being attached 10 cm proximal to the lateral malleolus and held in place with an inelastic strap that was looped around the therapy bed and fastened. Strap length allowed for an isometric contraction to be performed with the knee at 90° of flexion during testing. The participants were instructed to extend their leg for 5 s. Strong verbal encouragement was provided to ensure maximal effort. To provide moment value (Nm), the lever arm (length of femur or tibia) between the knee joint and the HHD was manually measured and subsequently normalized to their mass (Nm/kg). The averaged value of 2 measurements was used in the statistical analysis. Strong verbal encouragement was provided to ensure maximal effort.

### Sample size

The sample size calculation was made using the G*Power 3 program (Heinrich-Heine-Universität Düsseldorf, Düsseldorf, Germany) [21] with a power of 80%, significance level of 0.05, and moderate effect size (*f* = 0.15) [22]. We obtained a sample size of 68 participants. To allow for a potential data loss of 20%, the required sample size was 82 participants.

### Randomization and blinding

The participants were randomly allocated to the multiple exercise group or the control group. Block randomization was used. An independent researcher who performed no other assessments performed this allocation. The information of this randomization remained concealed for the assessors until the outcome assessments were finished. To conceal the randomization, consecutively numbered sealed opaque envelopes were prepared by a researcher with no other study involvement. The envelopes were stored in a locked location and opened in sequence within each stratum to reveal the group allocations.

### Statistical analysis

To minimize any bias produced by similarities between the right and left knees of the same patients, only one knee per patient (index knee) was analyzed. The index knee was defined as the more painful knee in the past or present. If patients felt that their knees were equally painful, the index knee was randomly selected using a computer-generated permutated block randomization scheme [23].

Multiple regression analysis was performed with post-intervention outcome measures as independent variable and the multiple exercise or control group as dependent variables (0, control group; 1, multiple exercise group). Pre-intervention outcome measures were included as covariates.

Data analyses were performed using JMP Pro 12.2 (SAS Institute, Cary, NC, USA). Values of *p* < 0.05 were considered statistically significant.

## Results

**Fig. 1:**
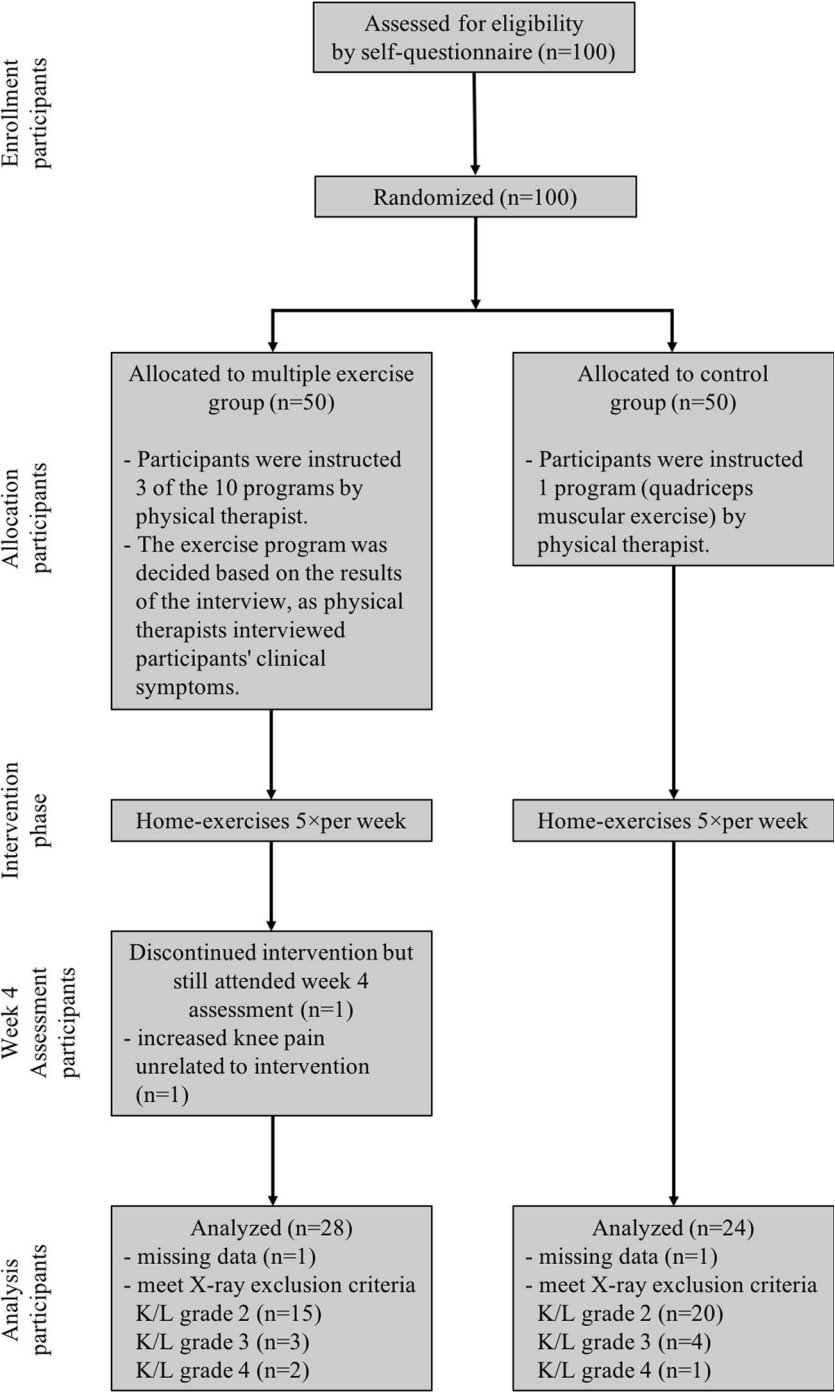
Flow diagram of the study protocol. X-ray, radiographic evaluation; K/L, Kellgren/Lawrence

A total of 100 participants were evaluated, of whom 45 (45.0%) were excluded due to the presence of mild radiographic knee OA (K/L grade = 2; *n* = 35 participants) or severe radiographic knee OA (K/L grade = 3 or 4; *n* = 10 participants), 1 (1.0%) was excluded because of discontinued intervention (increased knee pain unrelated to the intervention), and 2 (2.0%) were excluded because of missing data. Thus, a total of 52 participants were included (multiple exercise group, *n* = 28; control group, *n* = 24) in the final analysis (Fig. 1).

The baseline demographic and clinical characteristics for the 52 participants in each group are shown in Table 2.

**Table 2:** Demographic and clinical characteristics of the multiple exercise and control groups*

Variable	Multiple exercise group	Control group
	(*n* = 28)	(*n* = 24)
Age (years)	60.10 ± 6.99	58.33 ± 7.38
Women (%)	15 (53.6)	14 (58.3)
BMI (kg/m^2^)	23.88 ± 3.94	23.54 ± 4.31
Knee pain VAS (mm)	36.70 ± 16.38	36.49 ± 22.14
JKOM total (points)	16.96 ± 8.06	19.33 ± 11.74
Pain and stiffness (points)	7.53 ± 3.12	7.5 ± 4.86
Activities of daily living (points)	4.28 ± 3.55	3.25 ± 4.72
Participation in social activities (points)	3.60 ± 2.26	2.87 ± 2.25
General health conditions (points)	1.96 ± 0.99	2.0 ± 1.31
Strength of knee extension (Nm/kg)	1.09 ± 0.38	1.25 ± 0.53

*Values are mean ± SD or the number (percentage). VAS, visual analog scale; JKOM, Japanese Knee Osteoarthritis Measure

Table 3 shows the multiple regression analysis results. The multiple exercise group showed significant improvement in the prior intervention outcomes of knee pain on the visual analog scale, JKOM total, JKOM pain and stiffness, JKOM activities of daily living, JKOM participation in social activities, and JKOM general health conditions scores as well as knee extension strength measurements. The control group showed significant improvement in the prior intervention outcome of JKOM total score only. The JKOM activities of daily living and JKOM general health conditions outcomes improved significantly in the multiple exercise group compared to the control group (JKOM activities of daily living, beta = − 0.76; 95% CI, − 1.39 to − 0.13; *p* = 0.01; JKOM general health conditions, beta = − 0.25; 95% CI, − 0.48 to − 0.01; *p* = 0.03). In the JKOM participation in social activities outcome, an improvement trend was observed in the multiple exercise group compared to the control group (beta = − 0.37; 95% CI, − 0.77 to 0.03; *p* = 0.07).

**Table 3:** Multiple regression analysis results

Variable	Multiple exercise group (*n* = 28)			Control group (*n* = 24)			Differences in mean*	*p* value
	Pre	Post	Mean differences	Pre	Post	Mean differences		
Knee pain VAS (mm)	36.70 ± 16.38	21.32 ± 14.12	− 15.37 (− 23.57, − 7.18)	36.49 ± 22.14	26.85 ± 21.56	− 9.64 (− 22.34, 3.05)	− 2.77 (− 7.77, 2.22)	0.27
JKOM total	16.96 ± 8.06	9.85 ± 6.51	− 7.1 (− 11.03, − 3.17)	19.33 ± 11.74	12.79 ± 11.69	− 6.54 (− 13.35, 0.26)	− 1.26 (− 3.85, 1.32)	0.33
JKOM pain and stiffness	7.53 ± 3.12	4.71 ± 3.00	− 2.82 (− 4.46, − 1.18)	7.5 ± 4.86	5.91 ± 5.07	− 1.58 (− 4.47, 1.30)	− 0.61 (− 1.49, 0.27)	0.16
JKOM activities of daily living	4.28 ± 3.55	2.17 ± 2.53	− 2.1 (− 3.76, − 0.45)	3.25 ± 4.72	2.87 ± 5.25	− 0.37 (− 3.27, 2.52)	− 0.76 (− 1.39, − 0.13)	0.01
JKOM participation in social activities	3.60 ± 2.26	1.82 ± 1.46	− 1.78 (− 2.80, − 0.76)	2.87 ± 2.25	2.33 ± 1.71	− 0.54 (− 1.70, 0.62)	− 0.37 (− 0.77, − 0.03)	0.07
JKOM general health conditions	1.96 ± 0.99	1.14 ± 1.07	− 0.82 (− 1.37, − 0.26)	2.0 ± 1.31	1.66 ± 1.16	− 0.33 (− 1.05, 0.39)	− 0.25 (− 0.48, − 0.01)	0.03
Strength of knee extension (Nm/kg)	1.09 ± 0.38	1.46 ± 0.52	0.36 (0.12, 0.61)	1.25 ± 0.53	1.41 ± 0.52	0.15 (− 0.15, 0.46)	0.01 (− 0.13, 0.16)	0.82

Data are provided as mean ± SD and mean differences (95% confidence interval)

*These data are provided as beta (95% confidence interval) calculated on multiple regression analysis

For the multiple exercise group, the home exercise compliance rate was 96.6%. One participant reported an adverse event in week 3 (unrelated to the intervention) that increased the knee pain. In the control group, the home exercise compliance rate was 100%.

## Discussion

The purpose of this study was to investigate the effect of home exercise therapy aimed at improving the strength of multiple muscles and increasing joint flexibility in community-dwelling elderly individuals with pre-radiographic knee OA and examine patient adherence to the home exercise programs.

In the pre-radiographic knee OA subjects, significant improvements in knee pain, JKOM total and subscale scores, and knee extension strength were observed in the multiple exercise group, while significant improvements in JKOM total scores were noted in the control group. Moreover, the JKOM activities of daily living and general health conditions outcomes improved significantly in the multiple exercise group compared to the control group, while the JKOM participation in social activities outcome showed an improvement trend in the multiple exercise group compared to the control group. Patient adherence to both home exercise therapy programs was high.

In previous studies of the effect of a home exercise program on knee OA, home exercise has been reported to significantly improve knee OA-related pain and dysfunction in both surveillance and non-surveillance [24]. The results of this study supported those of the previous study.

The difference between the multiple exercise and control groups in this study was whether strength training of the hip muscles and stretching of the knee muscles was performed. Knee pain and dysfunction were significantly improved in the multiple exercise group; participants of which performed strength training of the hip muscles. In the previous study, improvement of the knee adduction moment was not observed by the strength training of the hip muscles for 12 and 8 weeks, whereas significant improvements in knee pain and dysfunction were observed [25, 26]. The knee adduction moment is an index for evaluating medial-to-lateral knee joint load that shows the effect of strength training on the knee joint [27]. One report stated that the knee adduction moment increases significantly since the extent of damage to the articular cartilage in patients with knee OA is stronger [28, 29]. There was reportedly no significant relationship in mild knee OA subjects between the knee adduction moment and knee pain and dysfunction [30]. Therefore, in patients with pre-radiographic knee OA (as in this study), the knee adduction moment is less involved in knee pain and dysfunction. For this reason, the fact that our study subjects were individuals with pre-radiographic knee OA could have contributed to the improvements in knee pain and dysfunction seen with the multiple exercise program.

Furthermore, knee extensor and hip muscles forces work cooperatively in the daily living movements (e.g., standing up, stair climbing) about which patients with knee OA complain of knee pain. In addition, patients with knee OA reportedly had significantly higher hip joint extension torque than the control group while standing up [31]. Therefore, patients with knee OA are expected to have excessive hip muscle activity to compensate for weakened knee extension muscle during knee joint loading. For this reason, in the multiple exercise group, improvements in knee pain and dysfunction occurred after load relief of the knee extension muscle was provided by strength training of the hip muscles.

Knee pain and dysfunction were significantly improved in the multiple exercise group; participants of which stretched the knee muscles. A previous study reported a significant relationship between ROM restriction of the knee and osteophytosis, bony enlargement, crepitus, and knee pain in subjects with early symptomatic knee OA [32]. Another study reported that an acute hamstring stretch improved knee extension range, peak torque, and stiffness in the final 10% of knee extension ROM [33]. Therefore, we thought that the improvement in knee pain and dysfunction was due to reduced knee joint stress and improved knee extension peak torque due to stretching of the knee muscles.

Significant improvement in knee extension strength was noted in the multiple exercise group. Previous studies reported that it takes 3–6 months for training effects to become evident regardless of age, sex, body size, or baseline muscle strength parameters in strength training of the knee extension muscles for treating knee OA [34]. For this reason, improvements to knee extension muscle strength in this study were not due to hypertrophy of the knee extension muscle. Other studies showed that knee extension strength decreased with strong knee pain regardless of K/L grade in patients with knee OA [35]. Accordingly, as knee pain improves, knee extension muscle strength improves. Therefore, the knee extension muscle strength improvements noted in this study were attributed to significant improvements in knee pain in the multiple exercise group.

The JKOM activities of daily living and general health conditions outcomes showed significant improvement in the multiple exercise group compared to the control group, while in the JKOM participation in social activities outcome, an improvement trend was observed in the multiple exercise group over the control group. The JKOM activities of daily living, participation in social activities, and general health conditions are used to evaluate physical functions related to activities of daily living and social functions including participation [19]. Because both groups conducted muscular strength training in the home exercise program, there was no difference in outcomes corresponding to knee OA-related pain and stiffness. In contrast, for activities of daily living, knee muscle strength and hip muscle function are both involved. Therefore, significant improvement was observed in the multiple exercise group; participants of which performed the hip muscle exercises.

High adherence to this home exercise therapy targeting strengthening multiple muscles and increasing joint flexibility was maintained. In this study, based on previous reports [15, 36], to improve participants’ understanding of the individualized exercise program, the participants were given an exercise instruction booklet and the exercise program was tailored to their individual situations. Moreover, previous studies reported that when subjects were contacted and supported during an intervention, high adherence to home exercise programs was maintained [37]. In this study, at week 2 of the intervention, we contacted the participants by e-mail and listened to their questions about the exercises. We believe that this component of the program contributed to the high adherence rate in the multiple exercise group.

This study has several limitations. First, our participants were motivated people because they responded to our program advertisements, which may have created selection bias. Second, self-reported questionnaires do not always provide precise data due to recall bias. Third, we did not include a control group not receiving treatment; as such, the symptomatic benefits observed may be related to the exercise environment and/or may have been an expectation of benefit rather than that of the exercise per se. Fourth, the number of enrolled participants did not reach the assumed sample size; thus, the effect size of the main outcome in this study may have been underestimated. Finally, all subjects of this study had pre-radiographic knee OA, so our results cannot be generalized to patients with mild or severe knee OA.

## Conclusions

This study aimed to investigate the effect of home exercise therapy to improve the strength of multiple muscles and increase the joint flexibility of community-dwelling elderly individuals with pre-radiographic knee OA and examine patient adherence rates. The multiple exercise group demonstrated significantly improved knee pain, JKOM subscale scores, and knee extension strength. Moreover, the JKOM activities of daily living and general health conditions outcomes showed significant improvement in the multiple exercise group compared to the control group, while the JKOM participation in social activities outcome showed an improvement trend in the multiple exercise group compared to the control group. A high adherence rate to both home exercise therapy programs was maintained. Our findings suggest that, when targeting community-dwelling elderly individuals with pre-radiographic knee OA, it is important to design and implement a home exercise program that aims to increase the strength of multiple muscles and improve joint flexibility rather than increase knee extension muscle strength only.
